# Artificial Intelligence (AI) Applications in Drug Discovery and Drug Delivery: Revolutionizing Personalized Medicine

**DOI:** 10.3390/pharmaceutics16101328

**Published:** 2024-10-14

**Authors:** Dolores R. Serrano, Francis C. Luciano, Brayan J. Anaya, Baris Ongoren, Aytug Kara, Gracia Molina, Bianca I. Ramirez, Sergio A. Sánchez-Guirales, Jesus A. Simon, Greta Tomietto, Chrysi Rapti, Helga K. Ruiz, Satyavati Rawat, Dinesh Kumar, Aikaterini Lalatsa

**Affiliations:** 1 Department of Pharmaceutics and Food Science, School of Pharmacy, Complutense University of Madrid, 28040 Madrid, Spain; fluciano@ucm.es (F.C.L.); branaya@ucm.es (B.J.A.); bongoren@ucm.es (B.O.); akara@ucm.es (A.K.); gramolin@ucm.es (G.M.); biancram@ucm.es (B.I.R.); sergsa16@ucm.es (S.A.S.-G.); jesimon@ucm.es (J.A.S.); greta.tomietto98@gmail.com (G.T.); xrisarapti1@gmail.com (C.R.); helgakar@ucm.es (H.K.R.); 2 Instituto Universitario de Farmacia Industrial, 28040 Madrid, Spain; 3 Department of Pharmaceutical Engineering and Technology, Indian Institute of Technology (BHU), Varanasi 221005, India; satyavatirawat1988@gmail.com (S.R.); dinesh.phe@itbhu.ac.in (D.K.); 4 Institute of Pharmacy and Biomedical Sciences, University of Strathclyde, 161, Cathedral Street, Glasgow G4 0RE, UK; 5 CRUK Formulation Unit, School of Pharmacy and Biomedical Sciences, University of Strathclyde, 161, Cathedral Street, Glasgow G4 0RE, UK

**Keywords:** artificial intelligence, AI, drug delivery, drug development, target identification, lead optimization, personalized medicines

## Abstract

Artificial intelligence (AI) encompasses a broad spectrum of techniques that have been utilized by pharmaceutical companies for decades, including machine learning, deep learning, and other advanced computational methods. These innovations have unlocked unprecedented opportunities for the acceleration of drug discovery and delivery, the optimization of treatment regimens, and the improvement of patient outcomes. AI is swiftly transforming the pharmaceutical industry, revolutionizing everything from drug development and discovery to personalized medicine, including target identification and validation, selection of excipients, prediction of the synthetic route, supply chain optimization, monitoring during continuous manufacturing processes, or predictive maintenance, among others. While the integration of AI promises to enhance efficiency, reduce costs, and improve both medicines and patient health, it also raises important questions from a regulatory point of view. In this review article, we will present a comprehensive overview of AI’s applications in the pharmaceutical industry, covering areas such as drug discovery, target optimization, personalized medicine, drug safety, and more. By analyzing current research trends and case studies, we aim to shed light on AI’s transformative impact on the pharmaceutical industry and its broader implications for healthcare.

## 1. Introduction

The adoption of AI in the pharmaceutical industry has evolved significantly over the past few decades. In the early stages, during the 1980s and 1990s, AI applications in drug discovery were limited to basic computational models, primarily used for molecular modeling and chemical structure prediction. These early efforts laid the groundwork for more sophisticated approaches as computational power and algorithms improved. By the early 2000s, AI began to gain traction with the introduction of machine learning algorithms capable of analyzing complex datasets, which helped streamline the drug discovery process by predicting molecular interactions and optimizing drug formulations. However, widespread adoption of AI in pharmaceuticals took off in the 2010s, driven by advances in Big Data, deep learning, and access to large biological and chemical datasets, such as those from genomics, proteomics, and high-throughput screening. Pharmaceutical companies started integrating AI into various stages of drug development, from target identification to clinical trial design. In recent years, AI has become an indispensable tool in accelerating drug discovery, optimizing clinical trials, and personalizing treatments, marking a shift toward more efficient, data-driven pharmaceutical research and development [1,2,3].

The convergence of artificial intelligence (AI) with the development of novel medicines has ushered in a new era of innovation which has significantly transformed several facets of drug discovery and drug delivery. AI has encompassed a wide range of techniques that have been applied by pharmaceutical companies over the last few decades, including machine learning, deep learning, and other advanced computational techniques. This has resulted in unprecedented opportunities for the expedition of the drug discovery and drug delivery processes, leading in turn to the optimization of treatment regimens and the improvement of patient outcomes [4,5,6,7,8,9,10,11,12].

Traditionally, the drug discovery pipeline has been characterized by high costs attributed to lengthy timelines and high failure rates. With the integration of AI-driven approaches, pharmaceutical companies can navigate this complex landscape more efficiently and effectively. For example, machine learning algorithms can analyze vast databases to identify intricate patterns. This allows for the discovery of novel therapeutic targets and for the prediction of potential drug candidates with better accuracy and at a faster pace than traditional trial and error approaches. This has sped up the drug development process for a myriad of diseases [13,14,15,16,17,18,19,20,21,22].

Similarly, AI algorithms can analyze large-scale biomedical data uncovering hidden relationships between drugs and diseases. This has allowed AI to fuel drug repurposing, facilitating the identification of new therapeutic uses for existing drugs and accelerating their clinical translation from bench to bedside. This is especially important for certain diseases such as parasitic diseases affecting developing countries as well as orphan diseases [23,24].

In the era of personalized medicines, AI algorithms can analyze diverse patient datasets, such as genomics, proteomics, and clinical records, and provide tailored treatments to individual patients based on their genetic makeup, lifestyle factors, and disease characteristics. This can minimize adverse effects and improve patient outcomes [25,26,27,28,29].

Even though the remarkable progress achieved so far is evident, the integration of AI into drug discovery and drug delivery is not without challenges. Ethical considerations, regulatory hurdles, and data privacy concerns continue to pose significant barriers to widespread adoption. A continued collaboration between researchers, clinicians, industry stakeholders, and regulatory bodies is pivotal to the powering of AI innovation in the pharmaceutical field [30].

**Figure 1:**
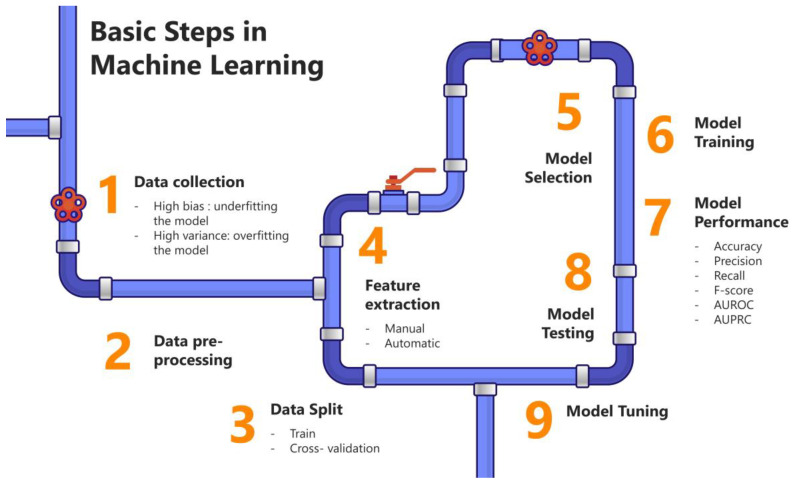
Schematic representation of the basic steps in machine learning classifier workflows.

An understanding of the basic steps involved in developing accurate and precise AI machine learning workflows is key before implementing them in pharmaceutical industrial processes (Figure 1). The first and most critical step is data collection and cleaning, as the quality of a model directly depends on the quality of the data it is trained on. To maintain data integrity, it is essential to inspect and correct any noise present, whether in non-image data (e.g., inaccurate entries, missing values) or image data (e.g., artifacts, uneven illumination). Additionally, the data should be reviewed for potential biases that might lead to underfitting, or high variance, which can cause overfitting. Overfitting occurs when the model learns patterns from noise or artifacts in the data rather than the true signal, resulting in poor generalization to unseen datasets with different biases. Techniques like cross-validation, expanding the training set, curating predictive features, and using ensemble methods can help mitigate the risk of overfitting.

Another important step in machine learning workflows is selecting and fine-tuning the optimal model based on its performance. Model performance is often evaluated using the Area Under the Receiver Operator Curve (AUROC), which measures the balance between sensitivity and specificity. Ideally, a good model should achieve high sensitivity and specificity, though the emphasis on one over the other may vary depending on the application. Generally, an AUROC greater than 0.80 is considered good, though the clinical acceptability of this threshold may differ based on specific use cases. However, there are limitations to relying solely on AUC. For example, while AUC measures the overall model performance in a population, it does not reflect confidence in individual predictions. In cases of class imbalance, where the positive class of interest is much smaller than the negative class, the Area Under the Precision-Recall Curve (AUPRC) may be a better performance metric than AUC.

After training and testing the model on a dataset—which is typically split into training and test sets—it is equally important to validate it on independent external datasets to ensure its stability and generalizability. Model development in AI is not a one-time process; the model needs to be periodically tested as new datasets become available. Regular maintenance is also required to ensure that performance remains robust, especially when faced with concept drift, which is where the relationship between input and output variables changes over time in unforeseen ways.

In this review article, we will provide a comprehensive overview of the applications of AI in the pharmaceutical industry, spanning drug discovery, personalized medicines, drug safety, and beyond. Through an analysis of current research trends and case studies, we aim to elucidate the transformative impact of AI on the pharmaceutical industry and its implications for healthcare delivery.

## 2. AI in Drug Discovery

**Figure 2:**
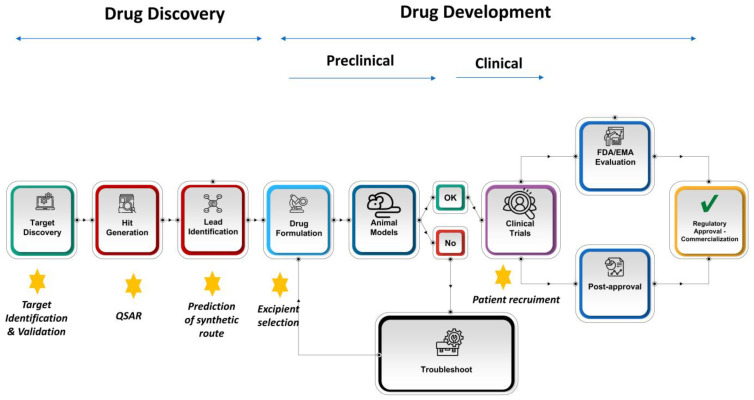
Schematic representation of the main stages during the drug discovery and drug development process. The star represents those stages where AI plays a key role in pharmaceutical processes.

Drug discovery is the process through which new pharmaceutical compounds are identified and developed for market release. This multi-stage process typically takes around 15 years to complete. The first step in drug discovery involves selecting a disease to focus on and identifying a target that may modify the disease. Next, exploratory research begins, during which large-scale screening tests help identify HIT molecules—chemical entities with promising affinity for the target. After further investigation, a molecule is chosen that binds specifically and selectively to the target and that can modify its normal mechanism of action. This molecule is called the LEAD compound. The lead compound is then optimized to enhance its biological activity and improve its ADME properties (absorption, distribution, metabolism, and excretion). If a promising compound is identified during screening, the drug moves into the preclinical (formulation studies and animal testing) and clinical phases. After the completion of clinical trials, the drug must be approved by regulatory bodies, such as the Food and Drug Administration (FDA) or the European Medicines Agency (EMA), before it can be marketed. Once on the market, the drug’s safety will continue to be monitored through pharmacovigilance throughout its distribution (Figure 2).

One of the primary challenges in drug discovery is the vast chemical space that must be explored to identify potential drug candidates. Traditional methods for screening large compound libraries are labor-intensive, time-consuming, and often result in a limited number of hits. Nevertheless, AI-driven virtual screening approaches leverage machine learning algorithms that can rapidly sift through vast datasets of chemical compounds as well as predict their biological activity against specific drug targets. These algorithms can analyze structural features, physicochemical properties, and molecular interactions to prioritize compounds with the highest likelihood of therapeutic efficacy. This can accelerate the hit-to-lead process optimization significantly.

Additionally, AI algorithms have played a pivotal role in the novo design of drug molecules with enhanced potency and selectivity. AI can generate optimized molecular structures targeting a specific biological activity while matching specific pharmacological and safety profiles by harnessing deep learning models and generative adversarial networks (GANs) [31]. GANs can be particularly useful for optimizing molecular structures, as they can generate novel compounds that target specific biological activities while adhering to pharmacological and safety profiles, thereby accelerating the drug discovery process. GANs are a type of deep learning model designed to generate new data samples that resemble a given dataset and consists of two neural networks: the generator and the discriminator. The first network (the generator) creates new data samples by learning the distribution of the training data with the aim of producing outputs that are indistinguishable from real data. In the context of drug discovery, the generator can create new molecular structures that mimic existing compounds with desirable properties. The second network (the discriminator) evaluates the samples generated by the generator and distinguishes between the real data from the training set and the fake data created by the generator. During training, the generator continuously improves its ability to create realistic samples, while the discriminator becomes more skilled at detecting fakes. This adversarial process continues until the generator produces samples that the discriminator can no longer reliably differentiate fake data from real data [32].

Apart from accelerating the identification of lead compounds, AI technologies are transforming the current landscape on how to optimize new leads and rethink drug design. In the past, the chemical synthesis of novel compounds has relied on trial-and-error approaches to iteratively modify lead compounds enhancing their potency, selectivity, pharmacokinetic, and toxicokinetic profiles. Nevertheless, AI-driven predictive techniques, such as quantitative structure–activity relationship (QSAR) modeling as well as molecular docking simulations, have provided new insights into how to predict the biological activity of novel compounds with great accuracy. The main reason behind this fact is the vast chemical and biological datasets utilized to generate AI algorithms focused on elucidating structure–activity relationships, minimizing the cost and the time for lengthy experimental validation [33]. QSAR models rely on the principle that similar chemical structures exhibit similar biological activities. QSAR models utilize molecular descriptors, such as molecular weight, electronegativity, or hydrophobicity, to capture the essential features of the chemical structure that may influence its biological activity (e.g., binding affinity to a target receptor or toxicity) [34].

As an example, several AI-powered platforms for drug discovery, such as Atomwise [35,36] and BenevolentAI [37], are revolutionizing the current way of finding new leads by prioritizing specific drug targets with the highest likelihood of therapeutic success, thereby accelerating the drug discovery process and reducing the risk of failure in clinical trials. These platforms leverage machine learning algorithms to analyze diverse datasets, including genomic, proteomic, and clinical data to identify novel therapeutic targets and predict their druggability [38].

In the last decade, AI-driven drug discovery has yielded promising results across a wide range of therapeutic fields, such as neuroscience, infectious diseases, oncology, and rare disorders. For example, DeepMind’s AlphaFold algorithm uses deep learning principles to demonstrate remarkable accuracy in predicting protein structures, which brings valuable insights into protein–ligand interactions [39]. Currently, more than 200 million proteins have been identified, with many new ones being discovered annually. Each protein possesses a unique three-dimensional structure that dictates its function and role. However, determining the precise structure of a protein can be a time-consuming and costly process, often requiring years of work and substantial financial investment. As a result, scientists have only been able to study a small fraction of these proteins, significantly hindering research efforts in disease treatment and drug discovery. Unraveling a protein structure involves predicting the forces of attraction and repulsion that ultimately define a protein’s three-dimensional structure. There are several experimental approaches to determine protein structure, such as nuclear magnetic resonance or X-ray crystallography, but both require extensive trial and error, years of meticulous work, and expensive specialized equipment. The AlphaFold algorithm has untangled this challenge by predicting protein-folding and accelerating protein discovery [40].

**Figure 3:**
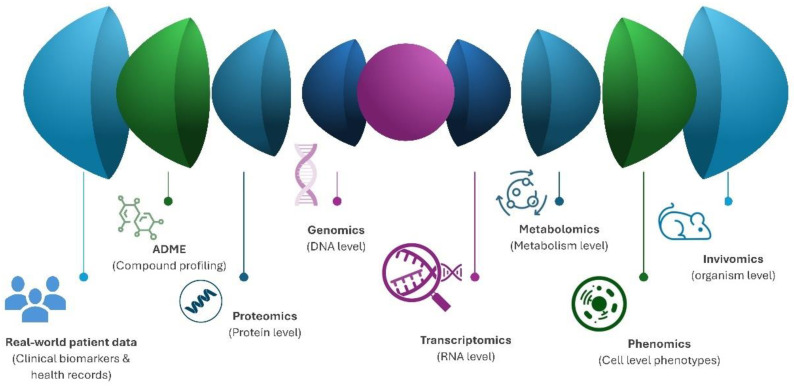
Schematic representation of Recursion Operating System algorithm.

Another example is Recursion, which also utilizes machine learning algorithms to screen thousands of compounds in parallel for the treatment of rare genetic diseases. In doing so, its aim is to accelerate the identification of potential drug candidates and foster their clinical translation [41]. Recursion is the proprietary of large biological, chemical, and patient-centric datasets (>50 petabytes) with more than 6 trillion gene and compound relationships profiled. Recursion’s central mission is based on the Recursion Operating System, a platform powered by one of the world’s largest proprietary biological and chemical datasets. Instead of looking narrowly at a handful of diseases with existing therapeutic hypotheses, the Recursion Operating System builds Maps of Biology and Chemistry that broaden the search to explore unknown areas of disease biology, integrating genomics, proteomics, metabolomics, transcriptomics, ADME (absorption, distribution, metabolism, and excretion) drug datasets, phenomics, invivomics, and real-world patient data (Figure 3). Recursion has several molecules in its pipeline, including an orally bioavailable small molecule superoxide scavenger developed for the treatment of central cavernous malformation; a novel small molecule designed to selectively inhibit the toxin produced by *Clostridioides difficile* in the gastrointestinal tract to prevent recurrent infections, which is a leading cause of antibiotic-induced diarrhea; and a CNS-penetrant, orally bioavailable, small molecule pan-Histone deacetylase inhibitor for the treatment of Neurofibromatosis- type 2-mutated meningiomas [42].

Despite the significant advances achieved in AI-driven drug discovery, several challenges remain unresolved. The interpretability of AI models, especially deep learning models, makes them complex and difficult to understand. This makes extracting the underlying mechanisms driving their predictions difficult, limiting their usefulness in guiding the rational selection of novel drugs. Moreover, a robust data infrastructure and specialized expertise are crucial elements for the integration of AI technologies into the drug discovery workflow. On top of this, ethical considerations, such as data privacy and algorithmic bias, should be taken into consideration to ensure a responsible and equitable use of AI in drug discovery [25].

## 3. Machine Learning in Drug Discovery

Machine learning algorithms have emerged as powerful tools in drug discovery, offering innovative solutions for virtual screening, target identification, and lead optimization. By leveraging vast datasets of chemical compounds, biological targets, and molecular interactions, machine learning algorithms can rapidly analyze complex relationships and predict promising drug candidates with enhanced accuracy and efficiency [43].

### 3.1. Virtual Screening

Virtual screening is the process by which large compound libraries are computationally screened for the identification of potential drug candidates. It is a critical step in the early stages of drug discovery. Traditionally, virtual screening methods were focused on molecular docking and pharmacophore modeling and were based mostly on rigid structures and simplified representations of ligand-target interactions that exhibited limited predictive accuracy. Nowadays, machine learning algorithms offer a more robust and flexible methodology for virtual screening, allowing for the analysis of a wide range of chemical features and the prediction of ligand-target binding with greater precision [15].

The main advantage of machine learning-based virtual screening is its capacity to learn complex patterns and relationships from large datasets of chemical compounds and biological targets. The success of a machine learning model is based on the training of its annotated datasets on known ligand-target interactions. Aiming a machine learning algorithm at this can allow it to identify subtle structural motifs and physicochemical properties associated with binding affinity, allowing for an accurate prediction of ligand-target interactions from novel compounds. Also, machine learning algorithms can bring together a wide range of information, such as protein structure data, gene expression profiles, the physicochemical properties of drugs, and drug-induced phenotypic changes, to improve the predictive performance of virtual screening models [44]. Among the most commonly used machine-learning approaches successfully applied to virtual screening are support vector machines (SVMs), random forests, and deep-learning models.

### 3.2. Target Identification

Identifying suitable drug targets is an important step during drug discovery as it establishes the biological pathway and molecular mechanisms that can be modulated to achieve therapeutic effects. Machine learning algorithms play a vital role in target identification. By analyzing diverse datasets of genomic, proteomic, and clinical data, these algorithms identify potential disease-associated targets and prioritize them for further investigation [45].

One of the main challenges in target identification is the vast amount of biological data available, including gene expression profiles, protein–protein interaction networks, and disease phenotypes. Machine learning algorithms offer a scalable and efficient approach to analyzing complex datasets and aim to identify patterns and associations that may not be apparent through traditional statistical techniques. By applying dimensionality reduction techniques, such as principal component analysis (PCA) and t-distributed stochastic neighbor embedding (t-SNE), machine learning algorithms can uncover hidden relationships between biological entities and identify potential drug targets based on their expression patterns, functional annotations, and disease associations [13].

Moreover, machine learning algorithms can integrate several sources of data to prioritize candidate drug targets based on their druggability, safety profiles, and therapeutic relevance. In this sense, the Drug Gene Interaction Database (DGIdb) uses machine learning algorithms to curate and annotate known drug–gene interactions from diverse sources, thereby allowing for the identification of drug targets from known interactions from approved drugs and experimental compounds [46]. The connectivity map (CMAp) also uses machine learning algorithms to analyze gene expression profiles from drug-treated cells and to identify potential targets based on their transcriptional signatures and functional annotations [47]. The connectivity map was developed to fill a gap related to the lack of methods available for systematically determining the cellular effects of a given compound as well as the unexpected off-target activities which may only be discovered late in the drug development process that could limit the compound’s clinical use. Based on this need, the connectivity map developed a comprehensive catalog of cellular signatures representing systematic perturbations with genetic and pharmacologic perturbagens. Signatures with high similarity might represent useful and previously recognized connections between two proteins operating in the same pathway, between a small molecule and its protein target, or between two small molecules of similar function but structural dissimilarity. Such a catalog of connections could serve as a functional look-up table for the genome [47].

### 3.3. Lead Optimization

Once potential drug candidates have been identified, lead optimization serves to improve their potency, selectivity, and pharmacokinetic properties through iterative chemical modifications. Traditionally, lead optimization relied on labor-intensive and time-consuming experimental approaches, such as high-throughput screening, which often resulted in suboptimal compounds and costly failures. Machine learning algorithms offer a more systematic and data-driven approach to lead optimization, allowing for the prediction of the biological activity and drug-like properties of novel compound analogs with greater precision and efficiency.

The application of machine learning-based lead optimization makes it possible to learn from large databases of chemical structures and biological activities to predict the structure–activity relationships (SARs) underlying drug-target interactions. By training predictive models on annotated datasets of known compound activities, machine learning algorithms can identify molecular features and substructures that contribute to the desired biological effects, guiding rational design decisions and minimizing the need for costly and time-consuming experimental validation. Among the machine learning approaches, QSAR modeling and GANs have gained in popularity. In this sense, the DeepChem framework uses deep learning algorithms to learn molecular representations directly from chemical structures and to predict the biological activities of new compound analogs with high accuracy [48]. Schrödinger’s Maestro platform performs molecular docking simulations and predicts the binding affinities of new compounds to target proteins and to prioritize lead candidates for further optimization [49].

By leveraging vast datasets of chemical compounds, biological targets, and molecular interactions, machine learning algorithms have shown great potential in rapidly analyzing complex relationships and predicting promising drug candidates with enhanced accuracy and efficiency. In Table 1, a summary of software platforms is provided.

## 4. AI in Predictive Modeling and Personalized Medicine and Formulation

### 4.1. AI and Personalized Medicines

#### 4.1.1. Prediction of Drug Responses and Optimization of Treatment Regimens

Machine learning and deep learning, particularly support vector machines, random forests, and neural networks, have become essential tools in predicting drug responses. More specifically, they have become essential tools for predicting how different patients will respond to specific drugs based on their unique biological characteristics . This can be integrated due to the vast amounts of biomedical data, including genomics, proteomics, and metabolomics, to identify potential biomarkers associated with drug efficacy and safety [53]. These models can guide clinical decisions on the most appropriate medications for patients, reducing the risk of adverse effects and improving overall treatment outcomes [54].

The optimization of treatment regimens can be also integrated within machine learning tools. By continuously learning from patient responses, AI algorithms can adjust dosing regimens in real-time, ensuring maximum efficacy while minimizing side effects. This has been applied to optimize chemotherapy dosing schedules in cancer treatments. AI can integrate data from multiple sources, such as electronic health records, clinical trials, and real-world evidence, to develop personalized treatment regimens. These plans are dynamic and are updated as new information becomes available, offering a more flexible and responsive approach to patient care [55,56].

The ability to identify patients who are most likely to benefit from a specific therapy can significantly reduce the risks of poor clinical outcomes and help lower treatment costs. This is particularly relevant for checkpoint inhibitor immunotherapies, where the overall response rates are relatively low (~20%), but certain patients experience exceptional, long-term benefits. Although the use of AI in this area has been limited due to data scarcity, it is gradually expanding [57]. Liu et al. developed a logistic regression-based classifier trained on genomic, transcriptomic, and clinical data from treatment-naïve patients to predict resistance to PD-1 inhibitors in advanced melanoma patients [58]. Johannet et al. introduced a more advanced AI method, using convolutional neural networks trained on histopathology slides along with patient clinical characteristics to predict checkpoint immunotherapy responses in advanced melanoma patients [59].

It is also crucial to quickly determine if a current therapy is ineffective for a patient so that clinicians can adjust or switch treatments in time. In clinical practice, cancer progression and treatment response are typically monitored by manually reviewing pathology or radiology images to assess tumor shrinkage and detect new lesions. However, this manual evaluation can be particularly challenging with checkpoint inhibitor immunotherapies, where disease progression patterns are often atypical [57]. To address this, Dercle et al. demonstrated the potential for using machine learning to train models on treatment-specific features to predict responses to different cancer therapies. They used an ensemble of six machine learning algorithms to predict patient sensitivity (defined as progression-free survival beyond the population median) to chemotherapy, targeted therapy, and immunotherapy by analyzing quantitative features from longitudinal CT scans of non-small cell lung cancer patients [60].

Beyond monitoring treatment responses, machine learning models like CURATE.AI offer dynamic options to adjust drug dosages for single or combination therapies, allowing for the tailoring of treatment to individual patients using time-specific data points [61]. Although cell lines can be imperfect models due to genetic drift or cross-contamination, they provide AI models with extensive data for learning. Pre-processing, such as cell line authentication or validation with in vivo data, is often necessary to minimize noise in these datasets. In one study, Iorio et al. assessed the response of 1001 cancer cell lines to 265 anti-cancer compounds to build Elastic Net models that translated genomic features, like mutations and gene expression, into drug efficacy predictions, thereby achieving accurate results [62].

A major limitation of AI learning models is their lack of interpretability regarding the biological mechanisms driving predictions. To overcome this, Kuenzi et al. developed DrugCell, an interpretable deep learning model that uses a visible neural network that mirrors known biological processes. By combining this with an artificial neural network designed to model a drug’s chemical structure, the model was able to accurately predict drug responses while also providing insights into the mechanisms driving the responses. Additionally, this approach was used to predict synergistic drug combinations and these predictions were able to be validated through patient-derived xenograft models [63].

#### 4.1.2. Tailoring Treatments to Individual Patients Based on Their Genetic Makeup, Lifestyle, and Other Factors

Personalized medicine is an approach that tailors medical treatment to the individual characteristics of each patient. One of the key applications of AI in personalized medicine is pharmacogenomics, the science focused on investigating how genes affect a person’s response to drugs. Making use of AI algorithms, a patient’s genetic makeup can be used to predict their response to different medicines, and hence can guide the selection of the most appropriate drug and dosage. For instance, AI models have been applied to predict patient responses to antidepressants based on genetic variation, helping clinicians to prescribe tailored psychiatric medications [64]. Identifying the most suitable antidepressant for a patient with a major depressive disorder is often challenging and it typically involves a trial-and-error approach. Machine learning offers a promising avenue for personalizing antidepressant prescriptions. However, while this is promising, its clinical utility remains limited and models need to be refined to consider other factors beyond effectiveness alone [65,66].

Beyond genetic information, AI also takes into consideration lifestyle factors, patient preferences, and environmental factors when tailoring treatments. Wearable devices and mobile health apps can collect real-time data on a patient’s physical activity, diet, sleep patterns, and other lifestyle factors. Analyzing all these data can provide insights into how these factors influence drug efficacy and disease progression [67,68]. Similarly, AI algorithms can incorporate social determinants of health, such as socioeconomic status, education, and access to healthcare, to provide a more comprehensive view of patient health. Bearing in mind all these factors, AI enables a holistic approach to personalized medicines, targeting customized treatments not only based on genetic factors but also taking into account the broader context of a patient’s life [69,70,71]. Machine learning models allow us to identify patient’s preference patterns as well. For example, a multivariate analysis detected a relationship between the pharmaceutical characteristics of ibuprofen tablets and patient preferences for those with shorter disintegration times and hence a faster onset of action [72].

### 4.2. AI in Formulation and Drug Delivery

**Figure 4:**
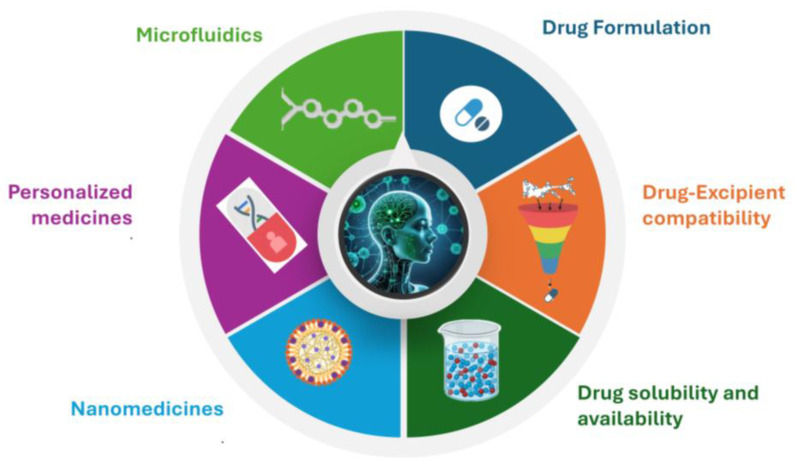
AI predictive modeling in personalized medicines, drug formulation, drug–excipient compatibility, drug solubility, bioavailability, nanomedicines, and microfluidics.

The pharmaceutical industry has long grappled with the complexities of drug formulation and delivery. Traditional methods often involve time-consuming and costly trial-and-error processes to optimize formulations and delivery mechanisms [73,74,75]. Predictive models generated by AI are applied to optimize drug formulations, ensuring that active ingredients are delivered to the target site in the body with maximum efficiency. For instance, AI algorithms can predict the release profile of a drug from a particular formulation, allowing for the design of controlled-release drug formulations that provide a steady therapeutic effect over time (Figure 4). Similarly, AI can also be applied in the design of drug delivery systems, including nanoparticles and liposomes, which can deliver drugs directly to specific cells or tissues. By predicting how these systems will interact with the body, the development of more effective and targeted drug-delivery technologies can be implemented [76,77].

#### 4.2.1. Optimization of Excipients and Drug Combinations and Compatibility

Excipients play a crucial role in determining the stability, bioavailability, and overall efficacy of pharmaceutical formulations. Traditionally, choosing the right combination of excipients involves extensive experimentation. AI-driven models, particularly machine learning algorithms, can analyze vast datasets to predict optimal excipient combinations that enhance drug performance. By building the correct dataset, AI models can successfully predict the optimal concentration of excipients required to achieve the desired disintegration and dissolution time [78,79].

The integration of AI into 3D-printed dosage forms has transformed pharmaceutical manufacturing, enabling personalized medicine and improving drug delivery systems [80,81]. The 3D printing of medicines allows a high versatility that cannot be achieved with conventional techniques, but its implementation in clinical practice is very challenging due to the complexity of fabrication and ensuring fine dose control without drug degradation [82,83,84,85,86]. AI algorithms can tailor the design and formulation of 3D-printed dosage forms to individual patient factors, such as age, weight, and medical history, resulting in customized drug therapies. AI can analyze extensive datasets and simulate the behavior of these dosage forms, facilitating rapid prototyping and the optimization of drug release profiles, dosage strengths, and geometries. Additionally, AI helps predict and address potential manufacturing challenges by optimizing printing parameters and ensuring quality control. AI-driven feedback systems further enhance the 3D-printing process by learning from real-time data, which improves accuracy, reproducibility, and scalability [87,88,89,90].

AI models can be also applied to understand and predict interactions between drugs and excipients which are key to ensuring stability and efficacy as well as preventing potential incompatibilities. Beyond real-time stability studies, conventional analytical tools, such as DSC, FTIR, NMR, and chromatography, are commonly used to detect potential drug–excipient interactions [91]. PubChem Fingerprint can be utilized as a dataset to represent the chemistry of both drugs and excipients comprehensively. DE-INTERACT is a machine-learning-based predictive tool for the study of drug–excipient interactions during product development [92]. Validation of the tool has been demonstrated through paracetamol and vanillin as a case study. The trained DE-Interact model achieved training and validation accuracies of 0.9930 and 0.9161, respectively. The model’s performance was validated by confirming three predicted incompatibilities using conventional analytical tools: paracetamol with vanillin, paracetamol with methylparaben, and brinzolamide with polyethyleneglycol. DSC, FTIR, HPTLC, and HPLC analyses confirmed these predictions [92]. Machine learning models can also be applied to the prediction of the critical quality attributes of solid dosage forms and their impact on the physicochemical performance of the formulation, depending on the manufacturing technique used [93].

#### 4.2.2. Enhancing Solubility and Bioavailability

The solubility and bioavailability of drugs are critical factors that determine their therapeutic effects. Drugs with poor water-solubility often face challenges in achieving sufficient bioavailability, as they may not dissolve adequately in the gastrointestinal tract, leading to reduced oral absorption and, consequently, lower therapeutic effectiveness. This issue is prevalent in nearly 40% of newly developed chemical entities, making it a significant hurdle in drug development

The prediction of drug solubility in aqueous media is a keystone during the earlier steps in the development process and can serve to guide the right solubilization strategy. In this case, machine learning models are trained on a large dataset of molecular properties and solubility data with the aim of identifying patterns that may not be apparent through conventional approaches [94,95,96]. Once a drug candidate is identified with a poor solubility profile, AI models are utilized to assist in the design of formulation strategies to enhance its solubility, such as solid dispersions, complexation, nanonization, and use of surfactants or co-solvents. Ultimately, bioavailability is the critical parameter influenced by several factors—not only aqueous solubility, but also, dissolution rate, permeability, and first-pass metabolism. Machine learning models can integrate data from in vitro, in vivo, and in silico studies to predict how a drug may behave in the human body, predicting its absorption rate and pharmacokinetic and bioavailability profile [97,98].

#### 4.2.3. AI in Designing Nanocarriers and Targeted Delivery Systems

Nanomedicines require the use of nanocarriers including liposomes, nanoparticles, dendrimers, polyplexes, transferosomes, and nano self-emulsifying systems, among others. Nanocarriers are used with the aim of targeting drugs to a specific region of the body at higher concentrations, thereby maximizing the drug’s efficacy and lowering its adverse effects on other areas [99,100,101,102,103,104]. This is of particular importance for targeting drugs towards cancer cells or infectious diseases, and also when triggering drugs with a poorly physicochemical profile through different physiological barriers such as the blood–brain barrier, stratum corneum, or intestinal epithelium [105,106,107,108].

The development of effective nanomedicine-based drug delivery systems is complex and requires the careful consideration of numerous factors, such as nanoparticle size, shape, surface change, and material composition, as these play a key role in the circulation time, cellular uptake, and biodistribution. For example, the smaller the size, the longer the circulation time with higher penetration in the deeper tissues, while rod-shaped or elongated particles might exhibit enhanced cellular uptake compared to spherical ones [108,109,110]. Conventional approaches to designing and optimizing nanomedicines are often labor-intensive and involve extensive experimentation. AI models come in as a transformative tool, streamlining the design, optimization, and delivery of nanomedicines. By training the model on experimental data, AI algorithms can identify nanoparticle designs that maximize tumor targeting and minimize off-target effects [111]. Also, AI models can predict the most effective ligand combinations by analyzing data on receptor expression patterns and focusing on those ligands with the greatest binding affinity, thereby improving precision and effectiveness [112].

#### 4.2.4. AI in Microfluidic Chip Design for Advanced Nanomedicine Fabrication

Microfluidic devices are miniature fluidic circuits designed to manipulate liquids at the nanoliter scale. The precise control of process parameters provided by microfluidics enables exceptional optimization of nanomedicine quality and encapsulation efficiency [80,113,114]. The integration of AI into the design and optimization of microfluidic chips has further accelerated advancements, allowing for enhanced performance, reduced development time, and cost-effective production. Machine learning models can predict the outcomes of microfluidic processes based on input parameters like flow rates, channel dimensions, and reagent concentrations. AI can accelerate Computational Fluid Dynamics simulations by predicting flow patterns and mixing efficiencies within microfluidic channels. Surrogate models can approximate complex simulations, drastically reducing computation time. Using experimental data, AI models can learn the underlying physics of microfluidic processes, enabling accurate predictions without explicit physical modeling. AI can generate innovative microfluidic architectures optimized for specific nanomedicine fabrication processes [115]. Ultimately, integrating AI with sensors on microfluidic chips allows for the real-time monitoring of nanomedicine fabrication, detecting anomalies, predicting failures, and adjusting parameters on the fly to ensure consistent product quality [116,117].

#### 4.2.5. Challenges and Future Directions

While AI offers promising avenues in drug formulation and drug delivery, several challenges have been encountered, including: (i) data quality and availability, considering that high-quality data and comprehensive datasets are essential for training effective AI models; (ii) model interpretability as AI models are complex and often function as “black boxes”, making it a challenge to interpret their decision-making process, this being essential for enhancing model transparency for regulatory acceptance and clinical trust; and (iii) regulatory considerations as the integration of AI into pharmaceutical development has already raised questions about the establishment of guidelines and standards for AI-driven methodologies to ensure safety and efficacy.

## 5. Examples of AI Applications in the Pharmaceutical Industry

AI is profoundly transforming the pharmaceutical manufacturing process, from the selection of excipients and synthesis route prediction to process optimization, drug design, supply chain, and preventive maintenance, amongst others. The use of AI in pharmaceutical companies has the potential to save significant amounts of both money and time across various stages of drug discovery and development.

AI accelerates hit identification, lead optimization, and preclinical testing by identifying compounds more quickly and accurately predicting their effects. AI-driven tools can streamline the drug discovery phase, which traditionally takes around 3–6 years. By predicting drug efficacy, toxicity, and optimal molecular structures more efficiently, AI can reduce this timeframe by 1–2 years. The cost of drug discovery can account for 35% of the total cost of developing a new drug, which averages around $2.8 billion [118]. AI can help cut drug discovery costs by reducing the number of compounds tested and improving the success rates of early-phase trials.

AI can also assist with the optimization of clinical trial designs, including patient recruitment, patient monitoring and reducing the length and expense of clinical trials. AI can also reduce the time needed to conduct clinical trials by automating data collection and analysis, allowing for a more efficient monitoring of patient outcomes. This has cut the length of trials by 15 to 30 percent [119]. By predicting adverse effects earlier and optimizing dosing strategies, AI can also cut down the time it takes for drugs to move from Phase I to Phase III. Molecules identified through AI have demonstrated higher success rates in early-stage clinical trials compared to those discovered via traditional methods. Phase 1 trials for AI-discovered drugs have achieved success rates between 80–90%, which is significantly higher than the historical industry averages of 40–65%. For Phase 2 trials, the success rate for AI-discovered molecules is around 40%, which is comparable to historical averages. It is expected that if these trends continue into phase 3 and beyond, the pharmaceutical industry could see an increase in the probability of a molecule successfully navigating all clinical phases from 5–10% to 9–18% [120].

**Figure 5:**
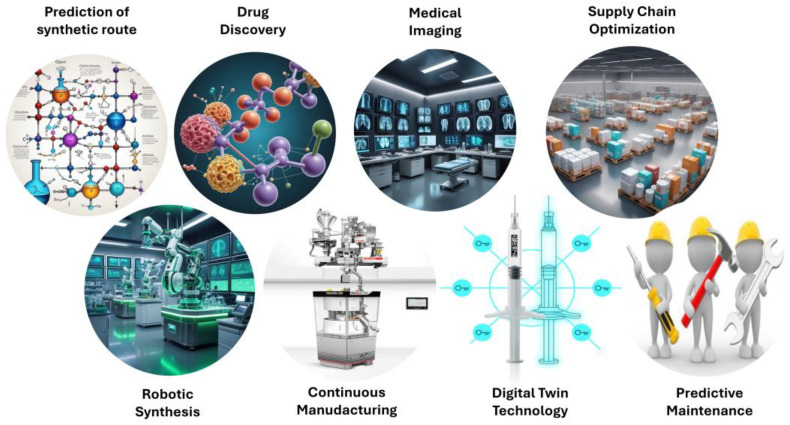
Examples of AI applications in the pharmaceutical industry. Image created using OpenArt.

Estimates show that the integration of AI can reduce costs and speed up processes in several ways, highlighting its potential to improve efficiency, reduce costs, and accelerate drug development (Table 2 & Figure 5). As AI technologies continue to advance, their integration into pharmaceutical manufacturing will likely become even more ubiquitous, driving innovation and improving patient outcomes.

### 5.1. Target Identification

The first step in the pharmaceutical company’s pipeline is to identify an accurate target. This process can be speed up with the use of AI tools. The Centre for Genomics Research at AstraZeneca will have analyzed up to two million genome sequences by 2026. With this vast dataset, AstraZeneca hopes to identify variants, genes, pathways, or other parts of the genome that are likely to cause disease and predict its progression and response to treatment. Using AI algorithms, new drug targets can be identified to design better medicines. This plays a key role in CRISPR gene-editing technology. Knowing what roles genes play in biology, CRSIP technology can be used to identify which genes when deleted lead to resistance or sensitization to cancer medicines. To get the most from every experiment, machine learning and deep learning models have been used to analyze the image-based outputs of CRISPR screens [121].

### 5.2. Drug Design

After the identification of a suitable target, AI algorithms can be applied in drug design by predicting the molecular structures and properties of potential drug candidates. By analyzing a vast biological dataset, machine learning models can identify druggable targets and design molecules with the ability to interact with the targets possessing the desired pharmacological properties. In silico Medicine is a biotechnological company that has developed a novel drug candidate for idiopathic pulmonary fibrosis in just 18 months using an in-house AI algorithm after screening billions of molecules and identifying a promising candidate that moved on into preclinical trials [50,122].

### 5.3. Compound Selection

Afterwards, AI algorithms can be applied to analyze large libraries of chemical compounds to identify those with the highest potential as drug candidates, including properties such as solubility, permeability, and toxicity. After predicting the drug structure, the candidate that would be the best can be determined, considering also its physicochemical properties for administration in the body. Exscientia is specialized in AI-driven drug discovery. They have designed EXS4318, a protein Kinase C-theta inhibitor that was licensed by Bristo Myers Squibb in 2023. PKC-theta plays a crucial role in controlling T-cell function, which is a major driver of autoimmune diseases. It is well known that PKC-theta inhibitors have potential in inflammatory and immunologic diseases. However, several large pharma companies have failed to design a small molecule with enough potency and selectivity against other closely related kinases. Exscientia’s AI algorithms have allowed them to design a highly potent and highly selective next-generation immunomodulatory drug candidate within just 11 months (150th molecule synthesized) [123].

### 5.4. Synthesis Route Prediction

Prediction of the synthetical route in pharma companies can be a tedious and long process before optimization. IBM has developed an AI-based retrosynthesis tool called “RXN for Chemistry”, which uses deep learning to predict chemical reaction pathways. This tool has been used by pharmaceutical companies to streamline the synthesis of complex molecules, reducing the time required to develop them. RXN for Chemistry uses AI to predict the outcomes of chemical reactions, retrosynthesis pathways, and experimental procedures based on molecular transformer models trained on 2.5 million chemical reactions. Molecular Transformer makes predictions by inferring the correlations between the presence and absence of chemical motifs in the reactant, reagent, and product present in the dataset. This class of models is flexible, non-rule-based, and scalable [124,125].

Two key issues arise in single-step retrosynthesis: (i) identifying the reaction center of the product, and (ii) generating the appropriate reactants and reagents once the reaction center is determined [126]. The first issue relates to replicating the decision-making process of a chemist when determining the disconnections in a target molecule. This task is difficult, as multiple decomposition pathways are often possible, and the best synthetic route depends on the overall structure of the route. Chemists rely on basic principles to intuitively prioritize bond breaking, but these rules are complex and poorly generalized across different products. For machines, a target molecule can have multiple reaction centers, allowing for various potential reactions, which leads to significant challenges for both model fitting and evaluation. The second issue involves identifying the necessary components for the reaction. Once the reaction center is defined, the target molecule can be broken down into synthons. Transforming these synthons into valid reactants and reagents is a complex task that must meet three levels of validity: (i) the generated reactants must adhere to correct chemical rules, so they must form valid molecules; (ii) the reaction from reactants to the product must be chemically feasible, considering factors such as the reaction center’s selectivity, the reagents used, reaction conditions, electronic effects, steric hindrance, and molecular orbital theory; and (iii) all atoms in the target product must map to those in the reactants, following the law of the conservation of atoms [127,128].

Additionally, the approach for generating reactants varies based on molecular representations; graph-based methods are prioritized for graph representations, while sequence-based methods align well with SMILES representations.

However, the ultimate goal of retrosynthesis planning is to generate a complete multi-step route. Fewer novel algorithmic efforts have been made to tackle the highly challenging task of multi-step retrosynthesis prediction that leads to commercially available building-block materials. Several key challenges arise in designing and evaluating effective retrosynthesis planning models. First, the search space for possible retrosynthesis plans is exponentially large, as each step toward the target molecule can be synthesized from hundreds of potential reactants. Second, the criteria for what constitutes a good synthetic route are often ambiguous and can vary depending on the context. For instance, in industrial settings, factors like stability and cost are prioritized, while in academia, the novelty and ability to tackle complex molecular structures are more important. Moreover, there are few publicly available datasets for retrosynthesis routes, and researchers often rely on hand-crafted routes to evaluate retrosynthesis algorithms. Therefore, AI-driven retrosynthesis planning is essential for accelerating route discovery in different contexts and automating the evaluation process [127,129].

### 5.5. Robotic Synthesis

Once, the synthetic route has been predicted and optimized, AI-driven robotics in pharmaceutical synthesis can automate the synthesis of chemical compounds, enabling high-throughput experimentation and faster drug discovery. Robotic systems when integrated with AI can carry out complex chemical reactions, monitor processes in real-time, and adjust parameters for optimal results. The University of Glasgow has developed Chemputer, a robot scientist guided by AI algorithms to automate the synthesis of drug molecules, which allows for a speeding up of the drug development process, making it useful tool in the synthesizing of small molecules [130,131].

### 5.6. Process Optimization

Once, the drug is formulated, the manufacturing process can be optimized to reduced cost and time using AI tools. AI algorithms can be used to optimize manufacturing processes by gathering data from production lines to identify inefficiencies and recommend improvements such as optimal reaction conditions, mixing processes, and the scaling up from lab to industrial processing. Pfizer implemented AI-driven process optimization in the production of its COVID-19 vaccine. By using AI to analyze data from the manufacturing process, Pfizer was able to improve yield and reduce production time, ensuring a steady supply of vaccines during the pandemic [132].

Pfizer also used machine learning algorithms to predict product temperatures and enable preventative maintenance for the over 3000 freezers that store the vaccine doses using sensors to track and monitor vaccine deliveries and temperatures with near-perfect precision. Additionally, supercomputing was used to run molecular dynamics simulations to identify the best mix of lipid nanoparticle features for decreasing allergic responses, resulting in a vaccination that was both safe and effective [133].

### 5.7. Continuous Manufacturing and PAT Technology

In contrast to batch manufacturing, continuous processes involve a constant flow of raw materials into the equipment, with the product being continuously discharged. The materials move through the system without interruption, eliminating any idle time between the various technological steps [134]. AI algorithms can be used to enhance several facets of pharmaceutical production from raw material sourcing to final product packing. This can ensure efficiency, cost-effectiveness, and high-quality output. Pharmaceutical companies have applied AI in continuous manufacturing processes for small molecules. AI systems can gather in situ information from probes connected in line, such as Raman or NIR, which monitor production parameters in real-time and make adjustments to maintain optimal conditions, resulting in a significant increase in manufacturing efficiency [135].

### 5.8. Digital Twin Technology

The manufacturing process can be easily replicated at different manufacturing sites using AI tools. Digital Twin technology, powered by AI, involves creating a virtual replica of the manufacturing process. This digital model mirrors the physical process in real-time, allowing manufacturers to simulate, monitor, and optimize without disrupting actual operations. Johnson & Johnson is leveraging digital twins to increase its speed to market. By testing products in one factory and utilizing a digital twin in another, the company can compare how the two manufacturing processes integrate [136].

### 5.9. Predictive Maintenance

AI-driven predictive maintenance involves analyzing data from equipment sensors to forecast when maintenance is required [137]. This approach helps prevent unexpected breakdowns by scheduling maintenance activities proactively. This application has reduced downtime and maintenance costs by accurately predicting equipment issues before they occurred. This approach is already implemented by many Pharma companies such as Pfizer [138].

### 5.10. Supply Chain Optimization

AI optimizes the pharmaceutical supply chain by predicting demand, managing inventory levels, and streamlining logistics. Machine learning models analyze market trends and performance data to ensure efficient supply chain operations. Novartis used AI to improve its supply chain logistics. By employing AI, Novartis enhanced inventory management and reduced operational costs, ensuring a more reliable supply of materials and products. The Buying Engine was designed to streamline and centralize purchasing decisions across Novartis, enhancing procurement efficiency. This algorithm-based platform serves as a “one-stop-shop”, initially focusing on lab supplies, PPE, and spare parts (indirect material). The system aims to provide transparency and recommend optimal purchasing options in near real-time by leveraging advanced techniques, such as knowledge representation, recommender systems, optimization, and machine learning algorithms [139].

### 5.11. Medical Imaging

For several years, Bayer has been using natural language processing, the technology behind large language models like GPT. This language plays a key role in medical coding, where the information collected by physicians in case reports must be translated into standardized terms and categories that can be analyzed and reviewed. To do this manually is a very time-consuming process. Bayer is the proprietor of a large language model that can process vast amounts of medical information with 96% accuracy and has currently processed millions of terms since 2017 [140]. Bayer is applying AI-driven technology in the field of radiology. Working with Blackford Analysis, a recently acquired imaging AI platform, Bayer has launched Calantic Digital Solutions which has been designed to support radiologists by automating time-consuming tasks, accelerating workflows, and enabling improved detection. The use of AI algorithms allows for a reduced workload and the delivery of faster decisions to patients [140].

**Table 2:** Examples that highlight the diverse applications of AI in the industrial manufacturing process of medicines and excipient selection, include synthesis route prediction, robotic synthesis, drug design, formulation optimization, compound selection, process optimization, data analysis, manufacturing optimization, process development, and excipient screening.

AI Application	Overview	Case Example	Reference
Synthesis Route Prediction	AI predicts optimal synthetic routes for APIs, analyzing chemical databases and literature to propose efficient pathways	IBM’s “Rxn for Chemistry” tool predicts chemical reaction pathways, used to streamline synthesis.	[124,125]
Robotic Synthesis	AI-driven robotics automate chemical synthesis, enabling high-throughput experimentation and faster drug discovery.	The “Chemputer” from the University of Glasgow automates drug molecule synthesis.	[130,131]
Drug Design	AI predicts molecular structures and properties of potential drug candidates, identifying druggable targets.	Insilico Medicine designed a novel drug for idiopathic pulmonary fibrosis using AI in just 18 months.	[50,122]
Drug Discovery	AI algorithms along with CRSIP technology enable the identification of which genes when deleted lead to resistance or sensitization to cancer medicines	AstraZeneca used AI to CRISPR gene-editing technology to identify new targets and make better medicines.	[121]
Compound Selection	AI analyzes chemical libraries to identify promising drug candidates based on properties like solubility, permeability, and toxicity.	Exscientia used AI to identify a novel compound for treating inflammatory and immunomodulatory diseases.	[123]
Process Optimization	AI optimizes manufacturing processes by analyzing data from production lines to identify inefficiencies and recommend improvements.	Pfizer used AI to improve yield and reduce production time for its COVID-19 vaccine manufacturing.	[132,133]
Continuous Manufacturing and PAT Technology	AI-driven optimization enhances various facets of pharmaceutical production, from raw material sourcing to final product packaging.	Pharmaceutical companies applied AI in continuous manufacturing, increasing efficiency.	[135]
Medical imaging	AI algorithms have been designed to support radiologists by automating time-consuming tasks, accelerating workflows, and enabling improved detection.	Bayer is using AI algorithms for reduced workload and delivering faster decisions to patients.	[140]
Digital Twin Technology	AI creates a virtual replica of the manufacturing process (digital twin) to simulate, monitor, and optimize processes in real-time without disrupting actual production.	Johnson & Johnson used digital twins to simulate and optimize their production processes, improving efficiency.	[136]
Predictive Maintenance	AI models analyze equipment sensor data to predict when maintenance is needed, helping to avoid unexpected breakdowns and schedule maintenance activities effectively.	Pfizer used AI for predictive maintenance in its manufacturing facilities, reducing downtime and maintenance costs.	[138]
Supply Chain Optimization	AI optimizes the pharmaceutical supply chain by predicting demand, managing inventory, and optimizing logistics based on market trends and performance data.	Novartis employed AI to manage supply chain logistics, leading to better inventory management and reduced costs.	[139]

### 5.12. Future Perspectives and Conclusions

AI is rapidly transforming the pharmaceutical industry, revolutionizing various facets from drug discovery, drug development, personalized medicines and many others. The integration of AI technologies promises to enhance efficiency, reduce cost and overall improve medicines and the health of patients, but at what cost?

The future of drug discovery is expected to be increasingly dominated by AI-driven approaches and will continue to advance, enabling more accurate predictions of drug-target interactions and a better understanding of disease physiopathology. AI models will be trained with larger biomedical datasets, including genomics, proteomics, metabolomics, and clinical trial information from patients to identify novel drug candidates as well as to optimize drug design reducing the risk of failure during clinical trials [4,13,141,142,143,144,145,146,147,148]. Additionally, AI has the potential to revolutionize clinical trials per se by improving patient recruitment, monitoring, and enhanced data analysis considering that advanced algorithms will enable the identification of suitable candidates based on genetic and phenotypic profiles ensuring that trials are conducted with the most appropriate cohort of participants [149,150,151,152,153,154,155,156,157,158,159].

AI will continue to drive the growth of personalized medicines by leveraging Big Data to tailor treatments to individual patients. Due to the ability to analyze genetic, environmental, and lifestyle data, the development of highly personalized treatment plans will continue to be widely implemented, addressing the specific needs of each patient [90,160].

AI-driven technologies will impact the pharmaceutical manufacturing processes which will benefit significantly from enhanced process optimization, quality control, and predictive maintenance, among others [161,162,163,164,165,166,167,168,169,170]. This implementation will allow for more efficient and scalable manufacturing processes, reducing costs and improving product consistency. AI-driven digital twins will simulate and optimize manufacturing processes in real-time, while predictive maintenance algorithms will prevent equipment failures and minimize downtime, facilitating more agile and responsive manufacturing operations [171,172,173].

Regarding AI-driven pharmacovigilance [174,175], AI will play a crucial role in improving drug safety by analyzing post-market surveillance data and identifying adverse drug reactions more efficiently, enabling faster responses to safety concerns and more informed decision-making regarding drug withdrawals or label changes [176,177]. Advanced natural language processing and machine learning models will extract valuable insights from electronic health records and social media, allowing for the detection and prediction of safety issues.

Finally, regulatory and ethical considerations will become by far more important as AI technologies become more integrated into pharmaceutical practices to ensure the transparency, fairness, and accountability of AI systems as well as maintain trust and compliance within the industry [178,179,180,181,182,183,184,185,186]. This makes necessary the implementation of regulatory frameworks that address the challenges associated with AI, including data privacy, algorithm bias, and the validation of AI-generated results.

For example, the HIPAA Privacy Rule in the U.S. sets forth national standards designed to safeguard the medical records of individuals and other identifiable health information, collectively referred to as “protected health information”. This regulation is applicable to health plans, health care clearinghouses, and healthcare providers who engage in specific electronic health care transactions [187]. This is aligned with initiatives such as the FDA’s Digital Health Innovation Action Plan that will continue to shape the regulatory landscape for AI-driven technologies in the pharmaceutical field in U.S., making sure that AI technologies are validated and used responsibly [188].

In Europe, the Commission published a draft regulation in April 2021 aimed at harmonizing standards concerning AI (AI Regulation) along with a coordinated plan that outlined a series of joint actions for the Commission and member states. This regulatory package aimed to enhance trust in AI and promote the development and advancement of AI technologies, emphasizing both the numerous social and economic benefits across various sectors and the necessity of safeguarding privacy while ensuring security and protection. The European Council adopted its position on the new AI regulations, advocating for a secure, lawful, and reliable AI that respects fundamental rights. The AI regulation in Europe was formally adopted by the Council on 21 May 2024, and came into effect on 1 August 2024 [189].

In conclusion, the incorporation of AI into the pharmaceutical industry is not just a technological advancement; it signifies a paradigm shift that could redefine global healthcare. The ongoing evolution of AI-driven drug discovery, clinical trials, and personalized medicine is expected to yield profound implications for patient outcomes, healthcare accessibility, and cost-efficiency. AI will continue accelerating drug discovery by enabling the rapid identification of viable drug candidates, which traditionally requires extensive resources and time. The ability of AI to analyze vast datasets quickly allows for the exploration of previously uncharted biochemical pathways and the design of novel compounds with targeted therapeutic effects. Moreover, AI’s capacity for real-time data analysis in clinical trials promises to improve patient recruitment and retention by predicting patient responses and lowering dropout rates. Such efficiencies not only boost the financial viability of drug development, but also pave the way for a more responsive healthcare system that can adapt to the needs of patients more swiftly.

Looking ahead, several trends are anticipated to shape the landscape of AI in pharmaceuticals: (i) the integration of AI with genomics, considering that genomic data will become increasingly more available and AI will play a pivotal role in tailoring treatments to individual genetic profiles, enhancing the efficacy of personalized medicine; (ii) AI-driven predictive analytics are expected to utilize AI for predictive analytics to forecast market trends, patient behaviors, and potential adverse effects, thus improving drug safety and efficacy; and (iii) regulatory adoption to accommodate AI technologies, ensuring safety and effectiveness without stifling innovation.

The long-term implications of AI in pharmaceuticals could be transformative for global healthcare. Enhanced drug development processes will likely lead to a faster introduction of novel therapies, addressing unmet medical needs more effectively. As AI optimizes resource allocation and improves operational efficiencies, it may also contribute to lowering drug prices, thereby enhancing accessibility for patients worldwide. Furthermore, as personalized medicine becomes more prevalent, treatment efficacy is expected to improve significantly, resulting in better health outcomes and a potential reduction in healthcare costs overall. This could alleviate some of the financial burdens on healthcare systems, particularly in developing countries where resources are limited.

In conclusion, the trajectory of AI in the pharmaceutical industry is poised to create a ripple effect across global healthcare, offering innovative solutions that enhance drug discovery, optimize clinical trials, and improve patient care. The collaborative efforts of stakeholders in this evolving landscape will be crucial for harnessing AI’s potential responsibly and effectively.

### 5.13. Use of AI Tools

Chat GPT (OpenAI, San Francisco, CA, USA) was used to compile information and to improve language readability. The full manuscript was carefully revised by the authors. Image F5 was created with the aid of OpenArt (Open AI, San Francisco, CA, USA). Figure 1, Figure 2, Figure 3 and Figure 4 were created with the aid of SlideModel (Montevideo, Uruguay).

## Figures and Tables

**Table 1:** Summary of software platforms that utilize AI techniques, such as deep learning, predictive modeling, and virtual screening, to accelerate various stages of the drug discovery and drug development process.

Software Platform	Description	Key Features	Ref.
DeepMind AlphaFold (Google, Mountain View, CA, USA) https://deepmind.google/technologies/alphafold/, accessed on 10 October 2024	Deep learning model for protein structure prediction	Predicts protein structures with high accuracy	[39]
Atomwise (Atomwise Inc., San Francisco, CA, USA) https://www.atomwise.com/, accessed on 10 October 2024	AI-driven drug discovery platform	Virtual screening, lead optimization	[36]
Recursion Pharmaceuticals (Recursion, Salt Lake City, UT, USA) https://www.recursion.com/, accessed on 10 October 2024	High-throughput screening platform	Cellular phenotypic analysis, rare diseases	[41]
BenevolentAI (Benevolent AI, London, UK) https://www.benevolent.com/, accessed on 10 October 2024	Drug discovery and development platform	Predictive modeling, target identification	[37]
Schrödinger Maestro (Schrödinger, New York, NY, USA) https://www.schrodinger.com/, accessed on 10 October 2024	Molecular modeling and simulations	Molecular docking, QSAR modeling	[49]
Insilico Medicine (Insilico Medicine, Hong Kong) https://insilico.com/, accessed on 10 October 2024	Drug discovery and biomarker development	Generative modeling, drug repurposing, and aging research	[50]
XtalPi (QuantumPharm Inc., Boston, MA, USA) https://www.xtalpi.com, accessed on 10 October 2024	AI-driven drug crystal prediction	Predicts drug crystal forms, stability	[51]
Cyclica (Cyclica, Toronto, ON, Canada) https://cyclicarx.com/science/, accessed on 10 October 2024	AI-driven drug discovery platform	Polypharmacology prediction, target deconvolution	[52]

## Data Availability

Data is available in the manuscript.
